# Investigating the Impact Behavior of Carbon Fiber/Polymethacrylimide (PMI) Foam Sandwich Composites for Personal Protective Equipment

**DOI:** 10.3390/ma17071683

**Published:** 2024-04-06

**Authors:** Xinyu Zhang, Miao Tian, Jun Li, Xinggang Chen

**Affiliations:** 1 College of Fashion and Design, Donghua University, Shanghai 200051, Chinatianmiao@dhu.edu.cn (M.T.); 2 Key Laboratory of Clothing Design and Technology, Donghua University, Ministry of Education, Shanghai 200051, China; 3 College of Materials Science and Engineering, North China University of Science and Technology, Ministry of Education, Tangshan 063210, China

**Keywords:** carbon fiber, energy absorption, impact resistance, polymethacrylimide, shock wave, ultra-high-molecular-weight polyethylene, personal protective equipment

## Abstract

To improve the shock resistance of personal protective equipment and reduce casualties due to shock wave accidents, this study prepared four types of carbon fiber/polymethacrylimide (PMI) foam sandwich panels with different face/back layer thicknesses and core layer densities and subjected them to quasi-static compression, low-speed impact, high-speed impact, and non-destructive tests. The mechanical properties and energy absorption capacities of the impact-resistant panels, featuring ceramic/ultra-high molecular-weight polyethylene (UHMWPE) and carbon fiber/PMI foam structures, were evaluated and compared, and the feasibility of using the latter as a raw material for personal impact-resistant equipment was also evaluated. For the PMI sandwich panel with a constant total thickness, increasing the core layer density and face/back layer thickness enhanced the energy absorption capacity, and increased the peak stress of the face layer. Under a constant strain, the energy absorption value of all specimens increased with increasing impact speed. When a 10 kg hammer impacted the specimen surface at a speed of 1.5 m/s, the foam sandwich panels retained better integrity than the ceramic/UHMWPE panel. The results showed that the carbon fiber/PMI foam sandwich panels were suitable for applications that require the flexible movement of the wearer under shock waves, and provide an experimental basis for designing impact-resistant equipment with low weight, high strength, and high energy absorption capacities.

## 1. Introduction

Since the 1990s, the number of terrorist attacks and industrial accidents, such as explosions, fires, and coal mine rock blasts, has surged. According to the World Health Organization, more than three million people die from impact injuries every year, mostly in middle- and low-income countries. Therefore, shock wave accidents pose a serious threat to public safety [1,2,3]. In certain high-risk environments such as war zones, riot scenes, and mine blast rescue operations, ordinary protective clothing is inadequate to shield the human body from shock waves. Therefore, wearing personal protective equipment (PPE) with high strength and shock resistance is essential for protecting the human body from impact [2,4].

**Figure 1:**
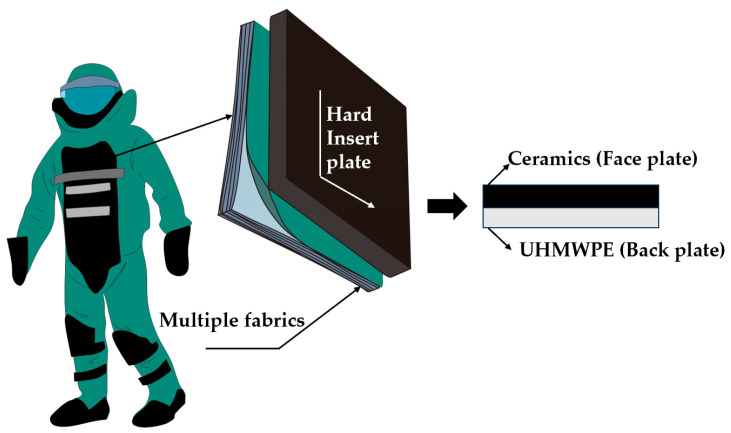
Basic structure of a bomb suit and hard insert plate.

Shock waves are high-pressure, high-speed waves that form when a bomb or industrial material explodes, instantly releasing enormous amounts of energy and rapidly expanding through various media. Studies on shock-resistant PPE have been ongoing for several years. Researchers want not only protection performance but also comfort features such as lightness and softness [5]. Because the performance of shock-resistant PPE is mainly affected by its constituent materials, the design and optimization of the composition and structure of shock-resistant materials have become a research focus for scholars. Bomb suits are common shock-resistant equipment that effectively protect wearers. However, their heavy weight (35–53 kg), low flexibility, and poor heat and moisture transfer cause severe fatigue in the wearer, owing to their outdated design and manufacturing [6,7]. Under the condition of low activity, the body heat production of the wearer increases by 3–4 times compared with the normal temperature status, and the metabolic rate of the human body increases by 49–78% during normal exercise. Many wearers have difficulty performing activities such as kneeling, jumping, squatting, running, and turning during the wearing process and experience heat stress, which can cause heatstroke, dizziness, confusion, and even life-threatening situations, significantly shortening the safe working time [8,9]. As shown in Figure 1, the main source of weight for the bomb suit is a hard insert plate made of bulletproof ceramics and UHMWPE laminate, which is designed to protect against fragment damage, but has limited protection against shock waves.

In disasters such as underwater explosions and coal mine accidents, shock waves cause more damage to the human body than explosion fragments, and rescuers need to have sufficient limb flexibility and lasting work ability while resisting shock waves. Most hard insert plates are too heavy, and their shock wave attenuation ability is inadequate, no longer meeting the work needs of rescuers who require flexible movement [10,11]. Therefore, there is an urgent need to develop insert plates that are safe, light, flexible, and cost-effective for strong shock wave protection and for protecting the lives of emergency rescuers.

In the 1980s and 1990s, researchers mainly used single-layer materials for blast protection and discovered that using foam materials, Kevlar, copper materials, or ceramic bulletproof vests alone worsened shock wave-induced damage to the gas-containing organs of the human body [12,13]. Several researchers studied the protection performance of copper/foam, Kevlar/foam, and ceramic–Kevlar composite materials and found that these composite materials can effectively protect the human body against shock-induced damage owing to impedance mismatching [14,15,16]. When shock waves propagate through a medium, the pressure acting on a certain area compared to the mass flow rate of particles passing perpendicularly through this area per unit time, which is the resistance, is called wave impedance. When foam materials are used solely as shock-resistant structures, the foam acts as a coupler owing to its wave impedance being lower than that of the human body. This increases the transmission coefficient, resulting in the enhanced transfer of stress waves from air to body tissues [13]. When copper or ceramic–Kevlar composite materials are added to the top of the foam layer, the human body is not affected owing to the decoupling effect of the high-impedance layer [14]. If the impedance of each layer of material is reasonably configured, it can reflect the incident wave to the maximum extent and reduce the transmitted wave. This phenomenon is also known as the impedance mismatch principle. This principle can be applied in the design of shock-resistant materials to reduce the direct stress coupled with the body, thereby lowering the severity of organ damage [14]. Currently, the widely used hard insert plate does not effectively exploit the impedance mismatching mechanism between different materials.

Sandwich composite materials originated from bionics, and are generally composed of a face, core, and back layer. In recent years, sandwich structures designed based on the impedance mismatching principle have been widely used in the fields of building and ship blast resistance. However, there is still room for improvement in the application of human body protection. A reasonable design of sandwich structures can reduce the mass load of PPE and enhance the protective effect on the human body, based on previous studies. If the materials are arranged in a high–low–high-impedance configuration to form a sandwich structure, the impedance mismatching principle can be used to attenuate the transmission coefficient, further attenuate the transmitted wave, and disperse and dissipate the impact load. In addition, using foam as the core material of the structure can make the structure lightweight with high strength and high energy absorption. In recent years, polymethacrylimide (PMI) foam sandwich panels have brought new ideas to the field of anti-impact protection owing to their excellent thermal resistance, low infrared, magnetic, and radar cross-sectional characteristics, and high shock wave resistance [17].

Early studies mostly combined PMI foam with aluminum panels and focused on the static mechanical properties of PMI foam sandwich panels [18,19]. Later, Yu et al. [20] conducted impact compression tests on aluminum-corrugated sandwich beams filled with PMI foam, demonstrating the advantages of filling a corrugated plate with PMI. Deng et al. [21] used a light gas gun to analyze the influence of PMI foam on the high-speed impact response of sandwich structures. Lai [22] reinforced glass fiber/PMI foam sandwich panels by stitching, and established a dynamic finite element model to predict their bending behavior. Du et al. [23] prepared carbon fiber/PMI foam sandwich panels based on origami structures. In the past decade, there have been studies on the mechanical behavior of PMI foam sandwich materials under impact and air blast loads. Although some researchers have investigated PMI foam sandwich structures for dynamic impact protection, most researchers still combine foam with metal panels, discussing their dynamic mechanical properties and applying them to protect ships and buildings against blasts. Thus, the performance of nonmetallic PMI foam sandwich panels for protecting humans against impact forces can be significantly improved further. However, designing lightweight, high-energy-absorbing, and wearable protective materials based on PMI foam sandwich structures remains challenging [24,25,26].

Thus, this study was conducted to strengthen the properties of shock-resistant PPE and reduce the damage caused by shock waves to the human body. We prepared four specimens of carbon fiber/PMI foam sandwich panels with low weights and strong energy absorption characteristics, and compared their impact behavior and energy absorption capacity with those of ceramic/UHMWPE hard insert plates. The results provided insights into the response mechanisms of the PMI foam sandwich materials to impact loads under different strain rates, and also revealed their feasibility as shock-resistant PPE insert materials. The insights gained from this research could be instrumental in the design and optimization of shock-resistant sandwich materials to meet environmental specifications, thereby propelling the progress of human impact protection equipment.

## 2. Materials and Methods

### 2.1. Theoretical Basis of Material Design

Wave impedance describes the resistance of a medium against shock wave propagation. It is defined as the ratio of the pressure exerted by the shock wave on a given area to the mass flux of the particles vibrating perpendicular to that area (i.e., the area multiplied by the particle vibration velocity). The value of the wave impedance is the product of the medium density *ρ* and the shock wave speed *C*. The reflection coefficient *F* and transmission coefficient *T* of the shock wave passing through the material are given as follows: they determine the intensity of the transmitted shock wave *σ_T_* and reflected shock wave *σ_R_* after the shock wave crosses the material boundary [7,27]. Here, “(*ρ*_0_*C*_0_)_1_” refers to the product of the density and wave speed of the first medium through which the shock wave passes, while “(*ρ*_0_*C*_0_)_2_” refers to the product of the density and wave speed of the second medium.

(1) $$\sigma_{T}=\frac{2}{1+\lambda }\sigma_{I}=T\sigma_{I},\;T=\frac{2}{1+\lambda }$$

(2) $$\sigma_{R}=\frac{1-\lambda }{1+\lambda }\sigma_{I}=F\sigma_{I},\;F=\frac{1-\lambda }{1+\lambda }$$

(3) $$\lambda =\frac{{\left(\rho_{0}C_{0}\right)}_{1}}{{\left(\rho_{0}C_{0}\right)}_{2}}$$

The final transmission coefficient of the material after passing through the 1st, 2nd, …, and nth layers in sequence is as follows:

(4) $$\begin{array}{ll} T & =\frac{2}{1+\frac{{\left(\rho_{0}C_{0}\right)}_{1}}{{\left(\rho_{0}C_{0}\right)}_{2}}}\cdot \frac{2}{1+\frac{{\left(\rho_{0}C_{0}\right)}_{2}}{{\left(\rho_{0}C_{0}\right)}_{3}}}\cdots \cdots \frac{2}{1+\frac{{\left(\rho_{0}C_{0}\right)}_{n-1}}{{\left(\rho_{0}C_{0}\right)}_{n}}}\\ & =\frac{2^{n-1}}{\left[1+\frac{{\left(\rho_{0}C_{0}\right)}_{1}}{{\left(\rho_{0}C_{0}\right)}_{2}}\right]\cdot \left[1+\frac{{\left(\rho_{0}C_{0}\right)}_{2}}{{\left(\rho_{0}C_{0}\right)}_{3}}\right]\cdots \cdots \left[1+\frac{{\left(\rho_{0}C_{0}\right)}_{n-1}}{{\left(\rho_{0}C_{0}\right)}_{n}}\right]}\\ & =\frac{2^{n-1}}{\prod_{i=1}^{n-1}\left[1+\frac{{\left(\rho_{0}C_{0}\right)}_{i}}{{\left(\rho_{0}C_{0}\right)}_{i+1}}\right]} \end{array}$$

The lower the overall transmission coefficient of the material, the stronger the attenuation effect of the material on the shock wave, and the better the protection performance. Therefore, the design of the hard insert plate of the sandwich structure should consider the impedance of each material layer. To reflect shock waves to the maximum extent, the face and back layers of a sandwich structure are suitable for hard materials with high acoustic impedance and toughness [28]. These materials are composed primarily of metals or polymers. However, metal materials often have the problem of being too heavy and having low bonding with the non-metal core layer. Therefore, in recent years, laminate materials composed of polymer fibers have become the main raw materials for the face layers of sandwich structures. The laminate materials currently used in explosion proofing and bullet proofing mainly include carbon fiber, aramid, UHMWPE, glass fiber, and basalt fiber [28]. Carbon fiber materials exhibit a high impedance, high strength-to-weight ratio, high stiffness-to-weight ratio, and low maintenance costs. They are also more compatible with harsh environments, such as high temperatures and under water, making them suitable for the face and back layer panels of shock wave protection inserts [27]. Some studies have suggested that unidirectional fabric composites exhibit a high impact velocity and energy absorption. Therefore, unidirectional carbon fiber fabric laminates are suitable for the surface and back layers of sandwich structures [29].

To achieve the maximum attenuation of the transmitted wave, low-impedance and high-energy absorbing materials are preferred for the core layer of a sandwich structure [30]. This material is generally porous and undergoes crushing deformation under an explosive/impact load, absorbing the explosive impact energy and effectively attenuating the shock wave and protecting the main structure [31,32]. PMI foam is a lightweight, 100% closed-cell rigid foam with the highest specific strength, specific modulus, and dimensional stability among most polymer foams. Unlike metal foam, PMI foam is compatible with various resins, and is isotropic and easy to machine into complex frame cross sections of different shapes. It also resists high temperatures and moisture, and can endure the high-temperature curing process of carbon fiber/epoxy and other materials. PMI foam is produced using a solid foaming process, which results in a high closed-cell rate, uniform and consistent pore size, and low moisture absorption. It can withstand static water pressure and is suitable for harsh environments, such as under water and high-temperature fires. According to the calculations, using PMI foam as the structural unit of the sandwich structure can reduce 1–2 layers of carbon fiber in the sandwich panel [17,33]. In addition to the core density, the face/core density ratio significantly affects the impact resistance of the sandwich panel. Tao et al. [34] indicated that under the same impact conditions, the optimal relative density ratio of the face and core layers for a sandwich panel is 2–5%, which provides the best overall energy attenuation effect of the material. In this study, we set the face layer panel density to 1300 kg/m^3^ and the core foam layer densities to 45 and 60 kg/m^3^.

**Figure 2:**
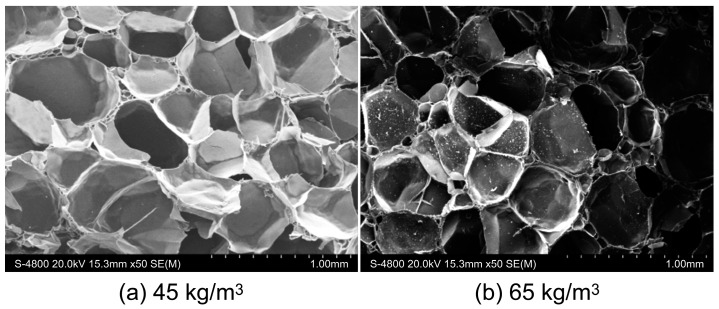
Microstructure of PMI foam.

To optimize the experimental parameters, we scanned the PMI foam using a Scios focused ion beam field emission scanning electron microscope (Scios, FEI Czech Co., Ltd., Brno, Czech Republic). Figure 2 shows that the PMI foam core layer had a high number of closed cells. The foam cells with a density of 45 kg/m^3^ were large, with an average diameter of ~533 μm, while those with a density of 65 kg/m^3^ were slightly smaller and exhibited a more uniform size, with an average diameter of ~466 μm.

### 2.2. Material Composition

As shown in Table 1, this study devised four types of sandwich-structured hard insert plates with varying parameters and produced parallel specimens for the control group based on the standard dimensions of conventional bomb suit chest hard inserts. To facilitate comparisons with the ceramic/UHMWPE bomb suit chest hard inserts, the total thickness of the sandwich panel specimens was set to 16 mm.

Sandwich panels were fabricated using a PMI foam material (WMI-PMI, Meiwo Engineering Materials Technology Co., Ltd., Baoding, China) and carbon fiber (T300)/epoxy woven prepreg (Zhongfu Shenyang Carbon Fiber Co., Ltd., Lianyungang, China) with a thickness of 0.25 mm per layer. The foam was first dried and dimensionally processed, and holes were evenly punched in the cut foam. Using an autoclave process, the carbon fiber prepreg was stacked in a [0/90] arrangement in a mold to form a laminate. To melt the resin and remove excess resin and gas, the laminate was placed back in an autoclave for preheating and prepressing. The melted resin flowed into the holes punched in the foam core layer, forming glue nails. The material was cured for one hour at 120 °C to solidify the resin.

**Figure 3:**
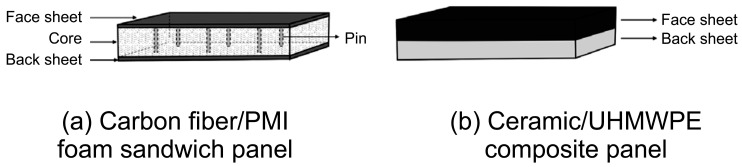
Structural diagrams of the two specimens.

Using a 400 D UHMWPE unidirectional fabric (Tessmann Special Rope Co., Ltd., Dongguan, China) and pressureless ceramic plates (Baile New Material Technology Co., Ltd., Yangzhong, China), we fabricated hard inserts for currently widely used bomb suits. The glass transition temperature of the UHMWPE is 145 °C, and it was cured for one hour at 120 °C to prepare a laminate. The UHMWPE laminate and the ceramic plate were processed to size and bonded using an epoxy resin adhesive. The structures of the carbon fiber/PMI foam sandwich panel and ceramic/UHMWPE composite panel are shown in Figure 3.

### 2.3. Quasi-Static Compression Test

To compare the responses of the carbon fiber/PMI foam sandwich panel and ceramic/UHMWPE composite panel under quasi-static loading, the material was tested using an INSTRON 5982 universal mechanical testing machine (Instron Corporation Norwood, MA, USA) following the standard ISO 844 [35]. The stress–strain curve of the specimen was obtained at room temperature at a compression rate of 1.25 mm/s.

### 2.4. Low-Speed Impact Test

The specimens were subjected to low-velocity impact tests using a drop-weight impact tester (Instron CEAST 9250), following the design of the hard plastic puncture performance test standard IS0 6603 [36]. The diameter of the drop hammer head was 16 mm, the impact mass was constant at 10 kg, and the appropriate impact energy was selected through a pre-test, which was finally determined to be 20 J. The drop hammer height was 150 mm and the impact speed was 1.5 m/s. The impact damage specimens and the corresponding stress–strain, contact force–time, and energy absorption curves of the five types of specimens were obtained experimentally. After the low-speed impact test, a Phoenix Vltomelxm-type three-dimensional X-ray computer tomography system was used to perform nondestructive testing on the post-impact specimens, and the internal damage of the sandwich specimens was analyzed.

### 2.5. High-Speed Impact Tests

The specimens were subjected to high-speed impact tests using a small-diameter split-Hopkinson pressure bar (SHPB). It is generally believed that the specimen size should be an order of magnitude larger than the pore size to reflect the true properties of the material. Considering the core layer micromorphology and the influence of the bar diameter on wave propagation, a pressure bar with a diameter of 16 mm was selected, and the specimen size was chosen as 15 mm × 15 mm × 16 mm. Owing to the impedance mismatching principle, the reflection and incident waveforms of the specimens were almost identical, whereas the transmission waveform was very small and nearly drowned by the interference signal. Therefore, to enhance the analysis of the experimental data, a paper rectifier was installed on the transmission rod. To detect the wave transmitted by the material, a strain rate of 1200 m/s was used in the experiments [37,38]. A bullet was fired by applying 0.15 MPa air pressure from an air compressor, which hit the incident rod, generating a constant amplitude pressure pulse in the incident rod. The pressure pulse propagated to the specimen along the incident rod, and part of it was reflected back, whereas the other part penetrated the specimen and continued to propagate forward along the transmission rod. The incident and transmitted pulses were recorded using an oscilloscope. Finally, the stress–time and stress–strain curves of the five specimen types were obtained. The energy absorption per unit volume, W, was computed by evaluating the area under the stress–strain curve.

## 3. Results

### 3.1. Quasi-Static Compression Performance

**Figure 4:**
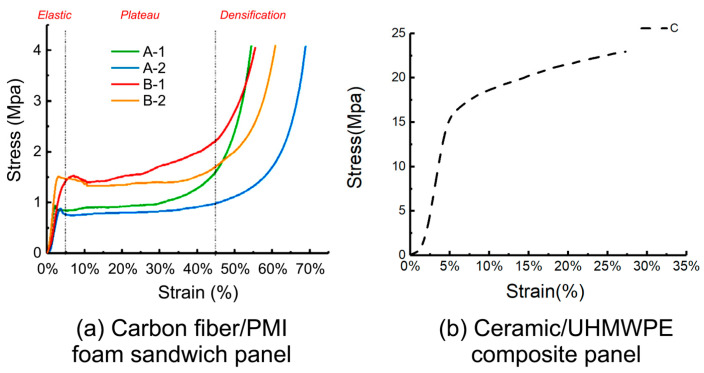
Stress–strain curve under quasi-static compression.

The typical stress–strain curves of the five specimens under quasi-static compression tests are shown in Figure 4. The stress–strain curves of the specimens showed three stages: elastic, plateau, and densification [39]. In a sandwich structure, the face layer provides bending and tensile strength, whereas the core layer enables large deformation under stress. Hence, the foam core layer mainly determines the stress–strain curves of the materials in quasi-static compression tests [40]. The PMI foam was evenly compressed and the cells deformed uniformly at the onset of the elastic stage. When the stress surpassed a certain proportional limit as the pressure increased, some cells began to deform plastically.

As shown in Figure 4a, the elastic stage lengths of the specimens were similar, occurring at the strain stage from 0 to 4%, and the 60 kg/m^3^ foam specimens (B-1, B-2) had a relatively longer elastic stage. This is because the extensibility of the foam edges and walls determines the elastic stage length. The foam with a density of 60 kg/m^3^ had smaller chambers, thicker foam walls, stronger ductility, and therefore a longer elastic stage [41,42]. The cell edges in the foam that deformed locally squeezed each other as the instrument loaded further, the cell walls bent and wrinkled, the foam partially collapsed, and the stress–strain curve slightly decreased. The curve rose and entered the plateau stage as the cells broke, the gas inside the cells escaped, and the pore gas became compressed. The material maintained a stable stress value while experiencing large-scale deformation at this stage, which shows that the foam material sandwich panel has good energy absorption capacity [40].

Figure 4a shows that the plateau stage occurred in the strain range of approximately 4–45%. The plateau stresses of curves A-1 and A-2 were similar, approximately 0.8 MPa, and the plateau stage of A-1 ended earlier than that of A-2. The plateau stresses of curves B-1 and B-2 were similar, approximately 1.5 MPa, and the plateau stage of B-1 ended earlier than that of B-2. The plateau stress of the B-type specimens exceeded that of the A-type specimens. This indicates that when the thickness of the sandwich panel is fixed, the plateau stress is strongly related to the foam density of the core layer. The higher the foam density, the higher the plateau stress. The length of the plateau stage is related to the foam thickness. When the face and back layers were thinner, the foam material was thicker and the strain amount of the compacted foam increased, resulting in a longer plateau stage. As compression continued, the curves entered the densification zone, where the load further compressed the foam matrix and the stress increased sharply [43]. After the test, the carbon fiber face and back layers of the specimens did not change significantly. It was difficult to observe cell rupture in the core layer with the naked eye, but there was an irritating gas overflow, confirming that the PMI foam had broken and released gas inside the cells.

As listed in Table 2, the Young’s modulus and yield strength of the materials were obtained from the quasi-static compression test results. The results showed that specimens A-1 and B-1, which had thicker face layers and smaller foam core ratios, had higher compressive strengths and compressive moduli than specimens A-2 and B-2, which had thinner face layers.

This indicates that increasing the face layer thickness and core layer density can improve the plateau stress and compressive modulus of the material, but shorten the plateau time.

By comparing Figure 4a,b, it can be observed that the stress endured by the ceramic/UHMWPE composite panel was several times that of the foam sandwich panel under the same strain. The linear elastic stage of specimen C was similar to that of specimens A and B. Subsequently, as the material was further compressed, the UHMWPE laminate exhibited fiber/matrix debonding and matrix cracking and entered the plateau stage. When the strain reached 35%, the instrument limit was triggered by excessive stress. The observation of the compressed specimens revealed that the ceramic layer was essentially undeformed, whereas the UHMWPE laminate layer was crushed. This indicates that the stress–strain curve of the ceramic/UHMWPE composite panel was mainly determined by the UHMWPE laminate layer. Because Specimen C was not completely crushed, its accurate compressive strength could not be obtained, and only its compressive modulus could be calculated. The compressive modulus of specimen C was much higher than those of specimens A and B.

### 3.2. Low-Speed Impact Performance

**Figure 5:**
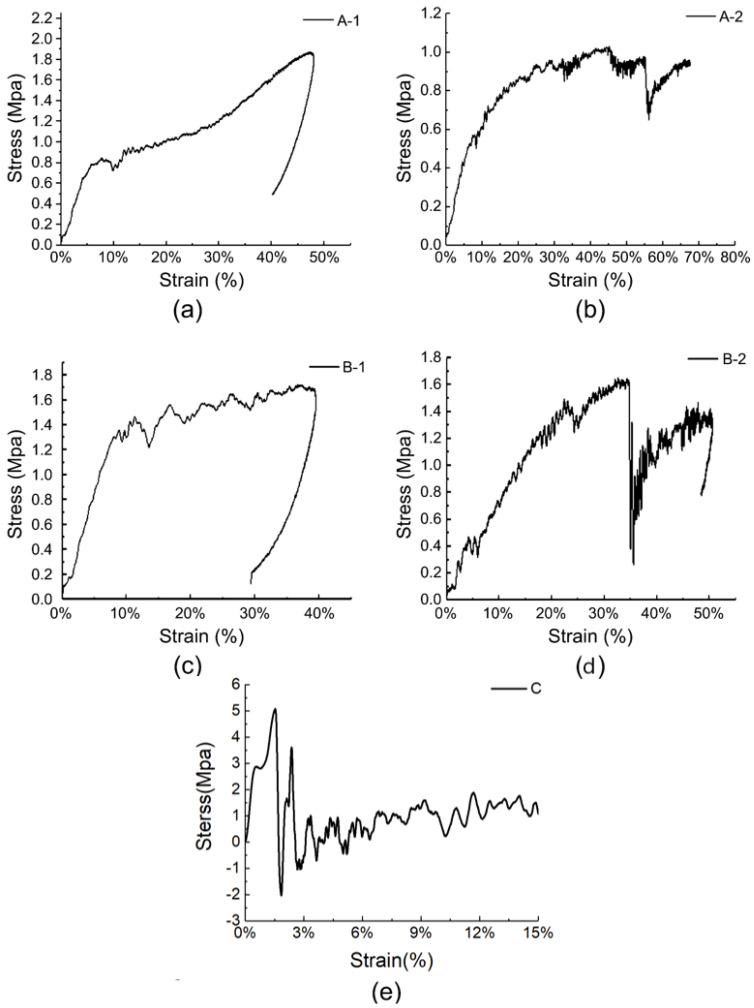
Stress–strain curve under low-speed impact: (**a**) A-1; (**b**) A-2; (**c**) B-1; (**d**) B-2; (**e**) C.

Figure 5 shows the stress–strain curves of the five specimens under low-speed impact tests. The curves reflect the processes of hammer loading and unloading stress from the contact with the specimens. As shown in Figure 5a,c, the loading and unloading parts of the curves of specimens A-1 and B-1 are smooth (the slight nonlinearity is caused by geometric nonlinearity during the impact process) without any slope change, indicating that no significant damage or cracking occurred in the face layer during the impact process.

However, in Figure 5b,d, the initial parts of the stress–strain curves of specimens A-2 and B-2 are linear, corresponding to the linear elastic behavior of the core foam before the face layer is damaged [26]. As the strain continued to increase, the stress suddenly decreased, indicating the cracking of the face panel. As the hammer continued to move downward, owing to the yielding plateau stage of the foam sandwich, the stress started to increase again and aggravated the cracking of the face panel until the instrument entered the unloading stage, and the stress decreased again. Figure 5e shows that the stress–strain curve of specimen C, the control group, manifested as a rapid drop after a brief rise in stress, followed by repeated fluctuations at a lower level, and the curve slope exhibited long-term and reciprocating changes. This phenomenon indicates that the specimen suffered severe cracking and damage after being impacted, and further damage occurred during subsequent loading; thus, no stress change occurred after the hammer moved away.

**Figure 6:**
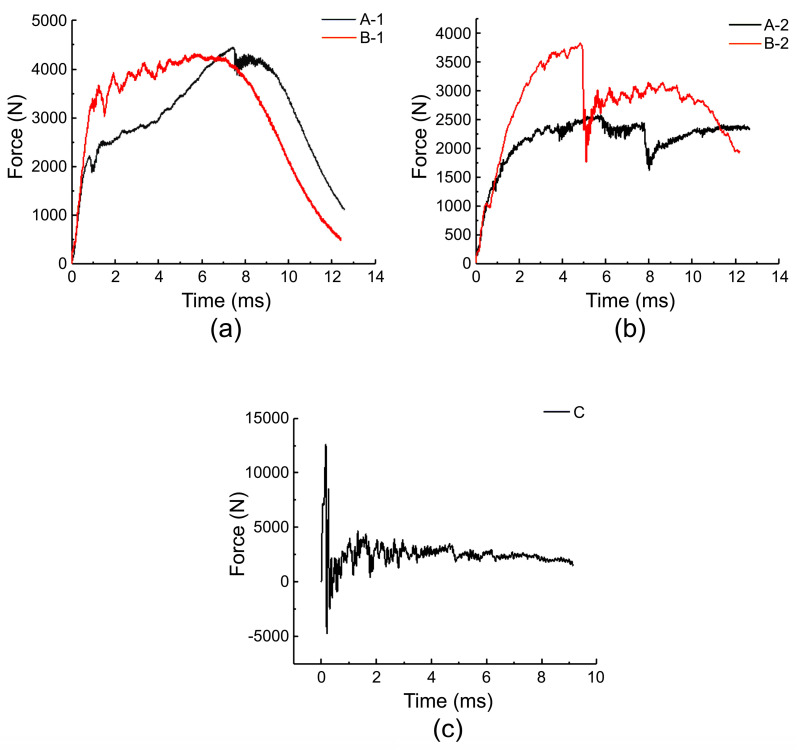
Force–time curve of specimens under low-speed impact: (**a**) force–time curves of specimens A-1 and B-1; (**b**) force–time curves of specimens A-2 and B-2; (**c**) force–time curves of specimen C.

Figure 6 shows the contact force–time curves of the five specimens, which reflect their mechanical response characteristics during the impact process. At the initial stage of the impact, the slope of the contact force–time curve is the largest, and the slopes of specimens A-1 and B-1 are higher than those of A-2 and B-2, indicating that the surface stress of the specimens with thicker face sheets increases faster. The difference in the peak impact load and occurrence time of the two groups of specimens in Figure 6a is small, indicating that changing the density of the core foam has little effect on the peak impact load of the specimens with the same face-sheet thickness, provided that the specimens do not show impact damage. However, the difference in the peak load and occurrence time of the two groups of specimens in Figure 6b is large, indicating that increasing the foam density leads to a higher peak load and shorter load occurrence time when the face sheet is thinner; this conclusion is consistent with the results reported by Feng et al. [44]. This is because as the foam density increased, the compressibility of the core layer decreased, and the plateau stress time decreased; thus, the peak load appeared fast, and the load value was high [24]. A comprehensive analysis shows that the order of the maximum load values of the five specimens was C → B-2 → A-2 → B-1 → A-1, and the peak impact load decreased in the order of C > A-1 > B-1 > B-2 > A-2. This indicates that for the same specimen thickness, the larger the core layer thickness, the lower the overall peak load of the specimen. When the specimen did not show any damage, the peak load did not change significantly with the foam density; however, when the specimen showed face sheet damage, the peak load increased with the foam density.

**Figure 7:**
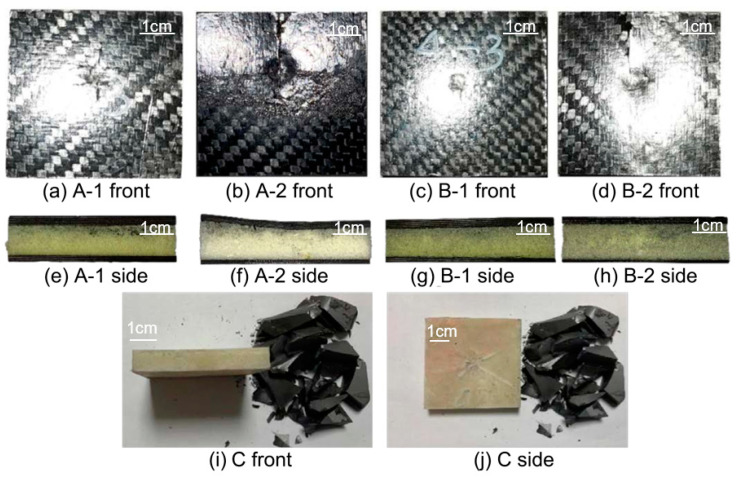
Images of five specimens damaged due to low-speed impact.

Impact damage images of the specimens are shown in Figure 7. The foam core layers of A-1 and B-1 had almost no change, and only slight dents appeared on the panel, with the average dent sizes of A-1 and B-1 being 0.41 ± 0.05 mm and 0.33 ± 0.09 mm, respectively. After being impacted by the hammer head, the panels of A-2 and B-2 both showed vertical cracks, and the deformation of the foam core layer could be observed from the side, with the average dent sizes of A-2 and B-1 being 0.62 mm and 0.77 mm, respectively. The damage to specimen C was specifically manifested as the crushing of the ceramic layer and the impact dent of the UHMWPE layer; the dent width of the UHMWPE layer was 17 mm, showing a star-shaped diffusion. However, no distinct side deformation was observed.

**Figure 8:**
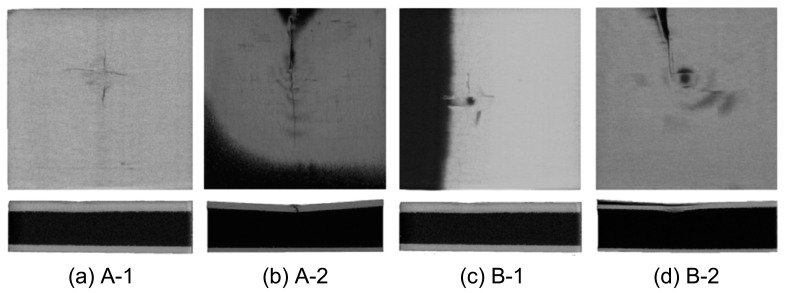
Computer tomography images showing the damaged face and core layers.

Figure 8 shows the damage to the face and core layers obtained using CT. In the scanned image, the cracking of the face layer panel can be observed more explicitly. The face layer of the material was damaged at the impact point, whereas the back layer remained intact. The face layers of specimens A-1 and B-1 were thicker, and the impact damage was smaller, which was reflected as a small cross-shaped dent, and small radial cracks appeared from the dent. The face layer panels of specimens A-2 and B-2 were thinner, and longer straight cracks appeared. Owing to the inclination deformation of the specimens, shadows appeared in the plane scanning images. From the perspective of core layer deformation, the core layers of specimens A-1 and B-1 hardly changed, whereas the panels of specimens A-2 and B-2 showed delamination and fracture, among which that in the central part hit by the hammer head was the most severe. The average dent depths of specimens A-2 and B-2 were 1.84 and 1.61 mm, respectively.

**Figure 9:**
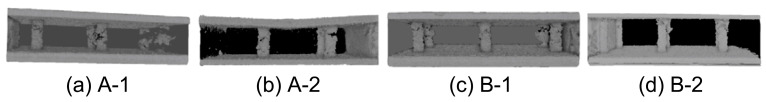
Images showing damaged glue nail.

Figure 9 shows the damage to the internal glue nails in the material. It can be seen that the deformation of the resin columns in the sandwich structure mainly occurred in the upper and middle parts. The closer the glue nails were to the middle position, the more severe the deformation, indicating that the resin columns helped bear a large compressive load in the longitudinal direction. The glue nail damage of specimens A-1 and B-1, with less panel damage, was stronger than that of specimens A-2 and B-2. This is because, when the material is subjected to a low-speed impact load, the impact head hits the upper panel, and the stress wave immediately spreads along the material in the horizontal and vertical directions. When the panel thickness is thicker and the core material thickness ratio is smaller, the glue nails have a faster and more sufficient response ability to disperse and absorb more impact energy through deformation. When the material panel is thinner and the core material thickness ratio is larger, the glue nails consume and absorb the impact energy. The deformation is not noticeable, and the stress at the core column is low; however, it increases the stress at the impact point, and thus the area damaged at the impact point in the upper panel expands, whereas only a small area of the glue nails is damaged [45].

In summary, it can be seen from the stress–strain analysis of the material, combined with the impact damage image, that the carbon fiber/PMI foam sandwich panel absorbs energy through the cracking of the panel and the deformation of the glue nail and foam when subjected to low-speed impact. When the impact load exceeded the bearing range of the glue nail and foam, the deformation mode of the internal glue nail manifested as microbuckling and breaking, and the glue nail breaking of specimens A-1 and B-1 with lighter panel damage was more serious. Specimens A-1 and B-1, with thicker panels, had lighter impact damage, whereas specimens A-2 and B-2, with thinner panels, exhibited delamination cracking of the panels and deformation of the core layer; the deformation degree of the core layer of specimen A-2 was the most serious. The damage situation of the specimens was highly correlated with the thickness of the face and back panels, and appropriately increasing the density of the core layer could further reduce the impact dent but would increase the peak stress of the material.

### 3.3. High-Speed Impact Performance

**Figure 10:**
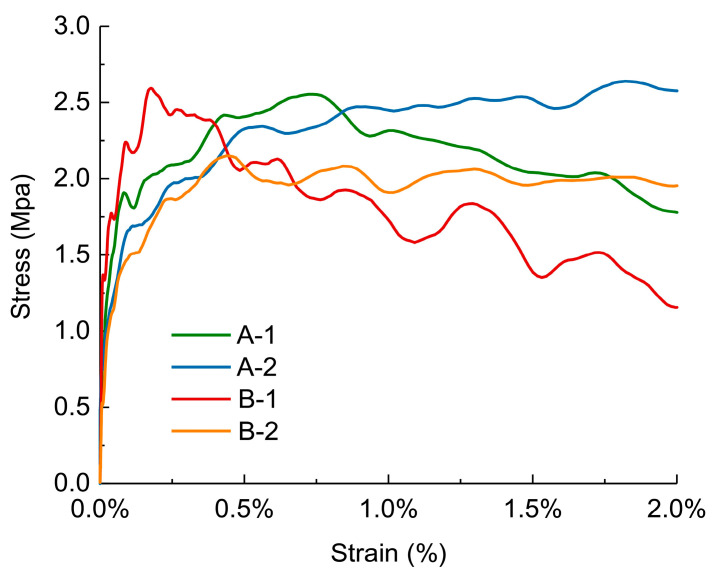
Stress–strain curves obtained under high-speed impact.

This section discusses the mechanical behavior of the four carbon fiber/PMI foam sandwich panels under high-speed impact. To facilitate comparisons, Figure 10 presents the stress–strain curves of the four foam sandwich specimens subjected to a strain rate of 1200 m/s in the first waveform cycle, where the oscillations of the curves are mainly attributed to the inertial effect. As observed in Figure 10, the stress gradually decreased after the deformation of each specimen surpassed 0.5%. Owing to the small strain, the stress–strain curve of the material terminated at the plateau stage. The overall stress of the A-type specimens was considerably higher than that of the B-type specimens, implying that, analogous to the low-speed impact, under the high-speed impact condition, when the thickness of the sandwich panel was identical, the core density was also the predominant factor influencing the shape of the stress–strain curve.

As can be seen from Figure 10, the specimens with a higher core density exhibited lower plateau stage stress. Moreover, when the core densities were equal, the curves of the two groups of specimens, A-1, A-2, B-1, and B-2, were not significantly different, indicating that the core thickness had a negligible effect on the waveform under this condition. Owing to the production constraints of the ceramic/UHMWPE specimens and the tolerance range of the Hopkinson pressure bar, we were unable to obtain the waveform curves of the ceramic/UHMWPE composite panel at the same strain rate.

**Figure 11:**
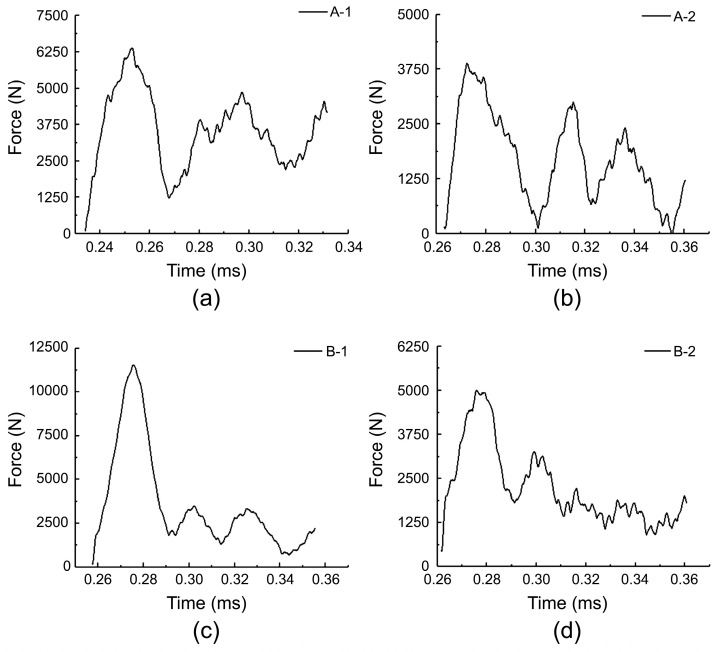
Force–time curves obtained under high-speed compression: (**a**) A-1; (**b**) A-2; (**c**) B-1; (**d**) B-2.

Figure 11 shows the stress–time curves of the four foam sandwich specimens under high-speed impact. As shown in Figure 11, the stress in each specimen varied periodically with time. Periodic oscillations were caused by the reflection and transmission of stress waves between the pressure bar, specimen, and transmission bar. Taking Figure 11a as an example, the bullet hit the incident bar, and after approximately 0.23 ms, the pulse propagated to the surface of the specimen and impacted the specimen, and the sandwich structure bore the load as a whole. Because the PMI foam was isotropic, the stress–strain curve exhibited a linear elastic rising stage. The specimen’s stress rose rapidly under the impact load, and the stress reached its peak at 0.25 ms, and the dynamic compressive strength of the PMI foam sandwich structure reached 2.54 ± 0.54 MPa. Subsequently, the local cells of the PMI foam started to collapse, and the stress exceeded the yield strength of the foam, causing the stress to decrease. The stress then fluctuated slightly around a certain value, which indicates hole wall damage, hole edge buckling, and pore gas compression of the single cells. The amplitude of the slight fluctuation is related to the energy absorption capacity of the PMI foam. Subsequently, the transmission bar reflected the shock wave again and a second pulse appeared. The sandwich specimen A-1 was further compressed and compacted after several impacts. At the end of the test, pungent gas overflowed, indicating that the foam cell wall had broken. As shown in Figure 8, the overall change rules of specimens A-1, A-2, B-1, and B-2 were similar. After the first pulse ended, the peak stress of each specimen decreased, and the group B specimens showed a more significant decrease. This shows that the specimens with higher core densities attenuated the incident waves more clearly. Among the four specimens, the peak loads of group B specimens were 11,458.4 ± 33.2 and 4951.8 ± 17.9 N, respectively, which indicates that under high-speed impact, a higher-density core layer also causes higher peak stress, similar to the low-speed impact condition.

## 4. Discussion

Over the past few decades, researchers have successively used foam sandwich structures to protect buildings and ships from impacts. In these studies, researchers generally evaluated the impact resistance of a material by evaluating its integrity and deformation degree after being subjected to impact, and inferred its performance as the main structure of the armor. However, when we use a material for human body protection, we need to make some adjustments to the evaluation indicators to better reflect the material’s protective effect on the human body structure. We found that the peak stress value and energy absorption capacity of the material surface are effective indicators of the protection capacity of the human body [46,47,48,49]. The lower the peak stress, the longer the stress rise time, the less compressive force applied to human organs. The stronger the energy absorption capacity, the better the protection performance of the material.

### 4.1. Peak Stress Value under Different Impact Velocities

In the quasi-static test, the stress–strain curves of the four carbon fiber/PMI sandwich panels were mainly related to the performance of the foam core layer. Due to the strong compressibility of the foam, the instrument triggered a limit when the stress reached approximately 4 MPa. In the plateau stage, we found that specimens B-1 and B-2 with a larger core layer density had higher stress, which indicated that the specimens with a larger density were more likely to densify under quasi-static conditions. The plateau stage stresses of the thicker B-1 and A-1 specimens were higher than those of the B-2 and A-2 specimens, respectively, indicating that increasing the thickness of the face and back panels improved the plateau stage stress under quasi-static conditions. In the low- and high-speed impact tests, we found that increasing the core layer density caused the peak stress value of the material surface to increase, whereas changing the thickness of the face and back panels affected the time of stress increase, and the specimens with thicker face panels had longer stress-rise times. In addition, we found that face layer damage had a significant impact on the peak stress value during the impact process. Damage to the face layer reduced the peak stress value of the material surface. Therefore, in the future, we can consider using the design idea of sacrificing the local face layer and increasing the overall protection performance of the structure when designing anti-impact equipment.

In general, the greater the peak stress value to which the specimen is subjected, the greater the risk of human organs being compressed. Therefore, when designing hard inserts, we should attempt to reduce the peak stress to which the specimen is subjected and extend the time of the stress rise. Notably, the peak stress of the four carbon fiber/PMI sandwich panels with different specifications was significantly lower than that of the ceramic/UHMWPE composite panels, and the stress increase was also gentle. This result implies that from the perspective of impact protection, carbon fiber/PMI sandwich panels can reduce the stress acting on humans to a greater extent and are more suitable as raw materials for anti-impact equipment. The face layer of the ceramic/UHMWPE composite panel had a higher hardness and poor deformation ability, which led to a rapid increase in the surface stress of the specimen. In the future, it can be modified or a rubber layer can be added between the ceramic and UHMWPE-laminated panels to extend the stress rise time of the material.

### 4.2. Energy Absorption Capacity under Different Impact Velocities

**Figure 12:**
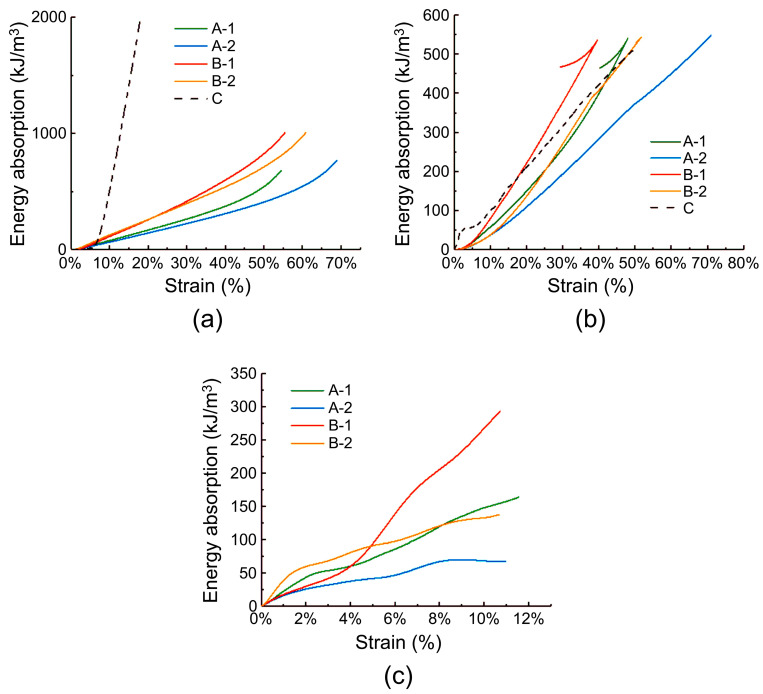
Energy absorption curves of the specimens: (**a**) quasi-static compression; (**b**) low-speed impact; (**c**) high-speed impact.

The energy absorption capacity is the characterization of the energy absorbed by a unit volume of a material compressed to a certain strain. The formula for calculating the energy absorption capacity per unit volume of the specimen is expressed as follows [50]:

(5) $$W=\int_{0}^{\varepsilon_{m}}\sigma d\varepsilon$$

where *σ* is the stress in the specimen, and *ε* is the strain. The original intention of designing a hard insert plate was to minimize the damage caused by the shock wave passing through the plate to the fabric structure and the human body behind it. This required the plate to absorb more energy within a certain stress range [51]. Figure 12 shows the energy absorption curves of the specimens under quasi-static compression, low-speed impact, and high-speed impact conditions. Evidently, under quasi-static conditions, the energy absorption capacity of the ceramic/UHMWPE composite panel is much higher than that of the carbon fiber/PMI foam sandwich panel, and among the sandwich specimens, B-1 with a thick panel and a core layer density of 60 kg/m^3^ exhibits the highest energy absorption capacity. Under low-speed impact conditions, the energy absorption capacities of the sandwich and ceramic composite specimens were equal, and under the same strain, B-1 had the best energy absorption capacity and the lowest deformation degree after impact. Because of the high hardness of the ceramic, it was difficult to achieve the same strain rate as that of the instrument; therefore, no comparative test of specimen C was performed under high-speed impact conditions. As shown in Figure 12c, under high-speed impact conditions, B-1 exhibited the strongest energy absorption capacity, followed by A-1. For ease of comparison, Table 3 lists the energy absorption levels of five types of samples when the strain reaches 10%. According to the table, the density of the carbon fiber/PMI sandwich panel is between one-sixth and one-quarter of that of the ceramic/UHMWPE composite panel, yet its energy absorption capacity under low-speed impact is comparable to that of ceramics, and the B-1 sample has the strongest energy absorption capacity under both low-speed and high-speed impact conditions. Combining Table 3 and Figure 12a–c, we can observe that the energy absorption capacity of the sandwich material increases with the increasing impact speed. According to previous studies, under the condition of a fixed total thickness, the thicknesses of the face and back layer panels and density of the core foam primarily affect the impact resistance of a foam sandwich panel [52,53]. The energy absorption results obtained in this study indicate that increasing the thicknesses of the face and back layer plates and foam density within a reasonable range results in an increase in the energy absorption value of the material under various impact speeds. In summary, under low- and high-speed impact conditions, the carbon fiber/PMI sandwich panel meets the requirements of protective equipment for lightness and high strength, has strong energy absorption capacity without significant damage and deformation, and is feasible for use as a hard protective insert plate.

## 5. Limitations and Future Outlook

Owing to technical constraints, there were significant discrepancies in the hardness and fabrication parameters between the ceramic/UHMWPE composite panel and the carbon fiber/PMI foam sandwich panel, which hindered us from conducting high-speed impact tests on them at the same strain rate and comparing their high-speed impact behaviors. Moreover, the impact tests of these two specimens were only performed at ambient temperature, and a performance comparison under high-temperature conditions is required in the future. To improve the toughness of the impact protection material, an aramid layer can be added in the preparation of the panel material, which can further enhance the attenuation ability of the sandwich structure to high-speed impact fragments, and the curved surface protection material can be prepared using the method of multi-element fragmentation design, which can minimize the preparation complexity and process costs of the foam sandwich impact protection equipment. In addition, to ensure that PPE wearers do not experience thermal stress and similar phenomena, sensor components can be incorporated into the design of the sandwich materials for the real-time assessment of the pressure conditions and of physiological states such as the heart rate and pulse of the wearer, to better define the safe working duration for the equipment users [54,55].

## 6. Conclusions

In this study, the impact behavior and energy absorption capacity of a carbon fiber/PMI foam sandwich panel were evaluated via quasi-static compression, low-velocity impact, and high-velocity impact tests, and the corresponding results were compared with those of a ceramic/UHMWPE composite panel. The results offer experimental support for developing hard-impact-resistant insert plates featuring low weight, high strength, high impact resistance, and strong energy absorption characteristics. In addition, the mechanism underlying the interaction between impact and composite materials was evaluated. The following conclusions were drawn from the findings of this study:

(1) Under quasi-static conditions, increasing the face sheet thickness and core density of the carbon fiber/PMI foam sandwich panels can improve the plateau stress and compressive modulus of the specimens, but shorten the plateau time.
(2) Under low-speed impact conditions, when the total thickness of the sandwich specimen remained unchanged, the larger the thickness of the core layer, the lower the overall peak stress value of the specimen. When the face layer did not show any damage, the peak stress did not change significantly with the foam density; however, when the specimen showed face sheet damage, the peak stress increased with the foam density.
(3) Under high-speed impact conditions, within a single waveform period, increasing the core layer density also increased the peak stress of the specimen, but appropriately reduced the stress level during the plateau period. The thicknesses of the face and back panels did not have a sufficient influence on the stress–strain curve of the specimen.
(4) Under an impact load, the carbon fiber/PMI foam sandwich panel can absorb energy through the cracking of the panel and deformation of the glue nail and foam. The deformation mode of the internal glue nail is manifested as micro-buckling and breaking. Specimens with thicker face layers and less damage exhibited faster responses and more severe breaking of the internal glue nails to the load. The impact integrity of the ceramic/UHMWPE composite panel was significantly lower than that of the form sandwich panel.
(5) From the perspective of human body protection performance, when the specimen thickness was the same, the energy absorption value of the foam sandwich material increased with an increase in the impact speed, core layer density, and panel thickness. B-1 and A-1 exhibited slightly higher energy absorption capacities than the ceramic/UHMWPE composite panel under low-speed impact.

Currently, PMI foam sandwich materials cannot be successfully applied in fields that require human body impact resistance. With a density of only 1/4 to 1/5 of the ceramic/UHMWPE composite panel, the energy absorption of the two kinds of composite panels is similar. In addition, the impact integrity of the sandwich panel is higher. Thus, the carbon fiber/PMI foam sandwich panel, which satisfies the design requirements of low weight and high energy absorption, can be utilized as a hard insert plate for PPE. Future studies should focus on further reducing the total thickness of the hard insert plate to further reduce its weight, while appropriately increasing the density of the core layer foam to control the peak stress and improve its energy absorption capacity.

## Figures and Tables

**Table 1:** Specimen alternatives and composition.

Alternatives	Composition	Thickness (mm^−1^)			Density (kg·m^−3^)	
		Face Layer	Core	Back Layer	Core	Specimen
A-1	Carbon fiber/PMI foam/Carbon fiber	3	11	2	45	454.47
A-2	Carbon fiber/PMI foam/Carbon fiber	2	13	1	45	321.54
B-1	Carbon fiber/PMI foam/Carbon fiber	3	11	2	60	492.23
B-2	Carbon fiber/PMI foam/Carbon fiber	2	13	1	60	330.08
C	Ceramic/UHMWPE	8	/	8	/	2053.43

**Table 2:** Compressive strengths and moduli of the prepared specimens.

Alternatives	Compressive Strength/MPa		Compressive Modulus/MPa	
	Mean	CV (%)	Mean	CV (%)
A-1	0.92	1.8	65.70	1.3
A-2	0.87	0.6	35.41	6.8
B-1	1.53	1.8	76.35	2.8
B-2	1.35	2.1	58.35	9.6
C	/	/	278.51	4.7

CV stands for coefficient of variation.

**Table 3:** Comparison of energy absorption capacity of the specimens.

Alternatives	Material Composition	Density (kg·m^−3^)	Number of Layers	Energy Absorption Value at 10% Strain (kJ/m^3^)					
				Quasi-Static Compression		Low-Speed Impact		High-Speed Impact	
				Mean	CV (%)	Mean	CV (%)	Mean	CV (%)
A-1	Carbon fiber/PMI	454.47	3	76.96	0.8	61.52	8.2	147.25	8.7
A-2	Carbon fiber/PMI	321.54	3	62.81	1.0	37.29	2.4	68.10	12.3
B-1	Carbon fiber/PMI	492.23	3	117.05	1.1	84.92	3.9	266.11	6.9
B-2	Carbon fiber/PMI	330.08	3	122.80	0.7	38.44	9.8	132.55	6.2
C	Ceramic/UHMWPE	2053.43	2	503.11	1.2	101.68	17.8	/	/

CV stands for coefficient of variation.

## Data Availability

The raw data needed to reproduce these findings cannot be shared at this time, as the data will be used in ongoing research.
